# Supplementary material for: Episodic construction of the early Andean Cordillera unravelled by zircon petrochronology
Source: Nat Commun. 2021 Aug 13;12:4930. doi: 10.1038/s41467-021-25232-z (PMC8363646; doi:10.1038/s41467-021-25232-z)
Supplement: Supplementary file 1 — Supplementary Information [file 41467_2021_25232_MOESM1_ESM.doc]

**Episodic construction of the early Andean Cordillera unravelled by zircon petrochronology**

José Joaquín Jara^1,2,3*^, Fernando Barra^1,3^, Martin Reich^1,3^, Mathieu Leisen^1,3^, Rurik Romero^1,3^, Diego Morata^1,3^

*(1) Departamento de Geología y Centro de Excelencia en Geotermia de los Andes (CEGA), FCFM, Universidad de Chile, Plaza Ercilla 803, Santiago, Chile.*

*(2) Departamento de Ingeniería de Minería, Pontificia Universidad Católica de Chile, Av. Vicuña Mackenna 4860, Santiago, Chile.*

*(3) Millennium Nucleus for Metal Tracing Along Subduction, FCFM, Universidad de Chile, Plaza Ercilla 803, Santiago, Chile.*

(*) Corresponding author: jjjara@uc.cl

**Keywords:** Cordilleran arcs; temporal evolution; episodic magmatism; zircon petrochronology

**SUPPLEMENTARY MATERIAL**

**Supplementary Methods**

**Samples and studied plutonic complexes.** Sampling was carried out in a transect north of the Huasco valley (~28°15’S) and along the range between 28°30’and 30°S. Fifteen whole-rock and 21 zircon geochronology and trace element analyses were performed in samples obtained from 10 different plutonic complexes. Petrological descriptions of the studied plutonic complexes and analysed samples are presented in Supplementary Table 1 and Supplementary Table 2.

Main mineralogical components of the samples are plagioclase, hornblende and quartz (Supplementary Table 2). Biotite could be present as a minor phase and pyroxene is usually replaced by hornblende. No evidence of deformation is observed. Alteration is minor and only represented by selective replacement of mafic minerals by chlorite, actinolite and/or epidote; and scarce albitization or sericitization of plagioclase was also observed in some samples.

Zircon grains selected for this study are inclusion-free, transparent and prismatic, with sizes ranging from 50 to 500 μm and a length to width ratio between 1.0 and 5.0. Cathodoluminescence (CL) images commonly reveal oscillatory zoning across the rims with homogeneous cores.

**Whole-rock analysis.** Determination of whole-rock major, minor and trace element concentrations were performed at the GeoAnalytical Laboratory, Washington State University, USA. Clean, unaltered rock chips (100 to 300 g) were ground in an agate mill. Concentrations of major and minor elements were determined by X-ray fluorescence with a single low dilution in a lithium tetraborate fused bead^1^. Trace elements were determined using ICP-MS with a di-lithium tetraborate low-dilution fusion followed by an open-vial mixed acid digestion^2^.

**Simultaneous zircon U-Pb geochronology and trace element analysis.** Five to 10 kg of sample were processed for zircon mineral separation by conventional comminution, magnetic and gravity procedures at the Department of Geology, University of Chile. Approximately 100 zircon grains per sample were handpicked and mounted in epoxy resin. Plešovice^3^ or SL2^4^ reference materials were used as a primary standard and SL2 or 91500^5^ as a secondary standard for geochronology purposes; USGS NIST 610 was used as a primary standard for trace element measurements. Polished mounts were analysis at the LEM-CEGA Mass Spectrometry Laboratory, University of Chile. Zircon crystal images (CL) were obtained by using a FEI Quanta 250 scanning electron microscope (SEM) coupled with a Centaurus sensor. Images were used to assess their internal structure. Simultaneous U-Pb geochronology and trace element concentrations were determined by laser ablation, inductively coupled plasma mass spectrometry (LA-ICP-MS). Analyses were carried out by using an Analyte G2 193 nm ArF excimer laser ablation system coupled to an iCAP-Q ICP-MS. Detailed analytical procedures and data reduction are described in ref. (6) and are briefly summarised here. Spot analyses (50 μm) were performed on the rims of the zircon grains. Each analysis considered 20 s of background followed by 50 s of data acquisition. The time-dependent drifts of U-Pb isotopic ratios and trace elements concentrations were corrected using a linear or exponential interpolation for every 5 and every 15 analyses, respectively. The reproducibility of the U-Pb geochronology was evaluated by comparison with the secondary reference material^5^. Off-line selection and integration of signals, time-drift corrections and quantitative calibrations were performed using Iolite^7^. Concordia diagrams and weighted mean calculations were constructed using Isoplot 4.0^8^. Uncertainties of individual analyses are quoted at the 2σ confidence level and include measured and propagated errors.

**Supplementary References**

1. Johnson, D.M., Hooper P.R., Conrey, R.M., 1999. XRF analysis of rocks and minerals for major and trace elements on a single low dilution Li-tetraborate fused bead. Advances in X-ray Analysis 41, 843-867.
2. Yu, Z., Robinson, P., McGoldrich, P.J., 2009. An evaluation of methods for the chemical decomposition of geological materials for trace element determination using ICP-MS. Geostandards and Geoanalytical Research 25, 199-217.
3. Sláma, J., Košler, J., Condon, D.J., Crowley, J.L., Gerdes, A., Hanchar, J.M., Whitehouse, M.J., 2008. Plešovice zircon - a new natural reference material for U-Pb and Hf isotopic microanalysis. Chemical Geology 249, 1-35.
4. Gehrels, G.E., Valencia, V., and Ruiz, J., 2008, Enhanced precision, accuracy, efficiency, and spatial resolution of U-Pb ages by laser ablation-multicollector-inductively coupled plasma-mass spectrometry: Geochemistry Geophysics Geosystems 9, Q03017.
5. Wiedenbeck, M., Allé, P., Corfu, F., Griffin, W.L., Meier, M., Oberli, F., Quadt, A.V., Roddick, J.C., Spiegel, W., 1995. Three natural zircon standards for U-Th-Pb, Lu-Hf, trace element and REE analyses. Geostandards Newsletter 19 (1), 1-23.
6. Liu, Y., Hu, Z., Zong, K., Gao, C., Gao, S., Xu, J., Chen, H., 2010. Reappraisement and refinement of zircon U-Pb isotope and trace element analyses by LA-ICP-MS. Chinese Science Bulletin 55 (15), 1535-1546.
7. Paton, C., Hellstrom, J., Paul, B., Woodhead, J., Hergt, J., 2011. Iolite: Freeware for the visualization and processing of mass spectrometric data. Journal of Analytical Atomic Spectrometry 26, 2508-2518.
8. Ludwig, K., 2010. Isoplot/Ex version 4.1, a geochronological toolkit for Microsoft Excel. Berkeley Geochronology Center, Special Publication 4.
9. Arévalo, C., Welkner, D., 2008. Geología del área Carrizal Bajo-Chacritas, Región de Atacama. Servicio Nacional de Geología y Minería: Santiago.
10. Welkner, D., Arévalo, C., Godoy, E., 2006. Geología del área Freirina-El Morado, Región de Atacama. Servicio Nacional de Geología y Minería: Santiago.
11. Emparan, C., Pineda, G., 2000. Área La Serena-La Higuera, Región de Coquimbo. Servicio Nacional de Geología y Minería: Santiago.
12. Arévalo, C., Mourgues, F.A., Chávez, R., 2009. Geología del área Vallenar-Domeyko, Región de Atacama. Servicio Nacional de Geología y Minería: Santiago.
13. Creixell, C., Arévalo, C., 2009. Geología del cuadrángulo El Tofo, Región de Coquimbo. Servicio Nacional de Geología y Minería: Santiago.
14. Arévalo, C., Creixell, C., 2009. Geología del cuadrángulo Tres Cruces, Región de Coquimbo. Servicio Nacional de Geología y Minería: Santiago.
15. Arredondo, C., Moscoso, R., Prieto, X., Ortega, R., Carrasco, R., Vivallo, W., Mateo, L., Pantoja, G., Ulloa, M., Ercilla, O., Ridelle, E., 2017. Depósitos minerales de la Región de Coquimbo. Servicio Nacional de Geología y Minería: Santiago.
16. Vivallo, W., Díaz, A., Jorquera, R., 2008. Yacimientos metalíferos de la Región de Atacama. Servicio Nacional de Geología y Minería: Santiago.
17. Middlemost, E.A.K. Naming Materials in the Magma/Igneous Rock System. Earth-Science Reviews 37, 215-244 (1994).
18. Irvine, T.N. & Baragar, W.R.A. A Guide to the Chemical Classification of the Common Volcanic Rocks. Canadian Journal of Earth Science 8, 523-548 (1971).
19. Peccerillo, A. & Taylor, S.R. Geochemistry of Eocene Calc-Alkaline Volcanic Rocks from the Kastamonu Area, Northern Turkey. Contributions to Mineralogy and Petrology 58, 63-81 (1976).
20. Frost *et al.* A Geochemical Classification for Granitic Rocks. Journal of Petrology 42, 2033-2048 (2001).
21. Shand, S.J. Eruptive Rocks. Their Genesis Composition. Classification, and Their Relation to Ore-Deposits with a Chapter on Meteorite (John Wiley & Sons, 1943).
22. Profeta, L. *et al.* Quantifying crustal thickness over time in magmatic arcs. Scientific Reports 5, [17786] (2015).

**Supplementary Table 1.** Geographic location, age and main characteristics of the studied plutonic complexes

| **Plutonic Complex** | **UTM Location** | | **General description** | **Main rock types** | **Reported age (Ma)** | **References** |
| --- | --- | --- | --- | --- | --- | --- |
|  | **N** | **E** |  |  |  |  |
| **Carrizal Bajo** | 6895000 | 295000 | Group of tabular plutonic bodies that are in contact with the Chañaral Epimetamorphic complex and the Algodones granite | Mg, Gdt (Bt + Amp)  Dt, Dqz (Px + Amp + Bt) | 208-206 | 9,10 |
| **Algodones Granite** | 6895000 | 305000 | Ellipsoidal, NE oriented pluton that is in contact with the Chañaral Epimetamorphic complex and Carrizal Bajo plutonic complex to the west | Mg, Gdt (Amp + Bt)  Dqz (Amp + Bt) | 203-197 | 9 |
| **Capote Granodiorite** | 6860000 | 304000 | Tabular and elongated, NE oriented homogeneous pluton that intrudes the Chañaral Epimetamorphic complex and Canto del Agua Fm. to the west and the La Negra Fm. to the east | Gdt (Bt + Amp)  Md, Dt (Bt + Amp + Px) | 190-183 | 9 |
| **Dioritic intrusions, north of La Serena city** | 6715000 | 278000 | A group of irregular bodies that outcrop in the coast north of La Serena city in a belt of 55 km. Intrude the Agua Salada Fm. | Mdqz, Dqz (Bt + Amp  + Px)  Md, Dt (Bt + Amp + Px) | 145 | 11 |
| **San Antonio Diorite** | 6890000 | 315000 | Ellipsoidal, NNE oriented homogeneous pluton that is in contact with the Chañaral Epimetamorphic complex to the west, Canto del Agua to the south, and La Negra Fm. to the east | Dt (Px + Amp) | 152-149 | 9 |
| **Infiernillo** | 6852000 | 307000 | An elongated, tabular body outcropping to the west of the AFS for more than 70 km in NNE orientation. To the west it intrudes the Chañaral Epimetamorphic complex and La Negra Fm., Punta del Cobre Fm. to the west | Dt (Px + Amp)  Gdt (Amp + Bt + Px) | 131-129 | 9,10,12 |
| **La Jaula Diorite** | 6866400 | 333000 | A group of irregular bodies of limited extension that intrude La Higuera plutonic complex to the south | Dt (Px + Amp) | 128 | 9 |
| **El Trapiche** | 6735000 | 295000 | Tabular, NNE elongated body composed of three tabular units. It intrudes the Punta del Cobre Fm. and is in contact with the Agua Grande plutonic complex to the east | Md (Opx + Clpx + Bt)  Dt (Opx + Clpx + Amp)  Mdqz, Gdt (Bt + Amp) | 121-117 | 13,14 |
| **Jilguero Granodiorite** | 6833000 | 339000 | Small, elongated, NS oriented body that intrudes the Punta del Cobre and Totoralillo Fms. | Gdt, Tn (Amp + Bt) | 93 | 12 |
| **Camarones** | 6818000 | 332000 | Composed, lenticular bodies of limited outcropping extension (8 km^2^), which intrude the Punta del Cobre, Nantoco and Totoralillo Fms. | Gdt (Bt + Amp)  Dt (Amp + Px) | 96-91 | 12 |

Notes: UTM coordinates according to Datum WGS84, Zone 19S. Main rock types from refs 9-16.

Abbreviations: Qz, quartz; Alkfs, alkali feldespars; Pl, plagioclase; Px, pyroxene; Amp, amphibole; Bt, biotite; Msc, muscovite; Opx, orthopyroxene; Clpx, clinopyroxene; Mg, monzogranite; Sg, sienogranite; Gn, granite; Gdt, granodiorite; Tn, tonalite; Dt, diorite; Gb, gabbro; Dqz, quartz diorite; Md, monzodiorite; Mqz, quartz monzonite; Mdqz, quartz monzodiorite.

**Supplementary Table 2.** Sample location, main characteristics and weighted average ^206^Pb/^238^U age for the studied plutonic complexes.

| **Sample ID** | **UTM Location**  **N E** | | **Plutonic Complexes** | **Lithology** | **Textures** | **Modal composition (%)**  **Qz-Kfeld-Plg-Px-Amp-Bt-Ms** | **Zircon U-Pb age (Ma)**  **(MSWD; n)** |
| --- | --- | --- | --- | --- | --- | --- | --- |
| JJJD_68  JJJD_69*  JJJD_65* | 6881928  6846509  6889940 | 288087  282040  294312 | Carrizal Bajo | Mg (Msc + Bt)  Dt / Dqz (Amp ± Px ± Bt)  Dqz (Amp + Bt) | Faneritic, inequigranular, euhedral to anhedral (~2 mm, up to 10)  Faneritic, inequigranular, euhedral to subhedral (~5 mm, up to 50)  Faneritic, inequigranular, euhedral to subhedral (~5 mm, up to 20) | 35-20-30-a-3-7-5  3-7-55-<5-30-<3-a  10-5-45-a-15-25-a | 215.7 ± 0.8  (0.77; 28)  210.0 ± 0.9  (0.20; 14)  208.0 ± 0.8  (0.99; 19) |
| JJJD_64 | 6887240 | 294983 | Algodones Granite | Mg (Amp + Bt) | Faneritic, inequigranular, euhedral (~5 mm, 30 to 5) | 35-18-30-a-7-10-a | 197.4 ± 0.7  (0.29; 43) |
| JJJD_71*  JJJD_73* | 6858886  6859399 | 304334  305264 | Capote Granodiorite | Gdt (Bt + Amp)  Gdt (Bt + Amp) | Faneritic, inequigranular, subhedral to anhedral (~3 mm, 10 to < 1)  Faneritic, inequigranular, euhedral to anhedral (~5 mm, 15 to < 2) | 20-5-40-<3-12-20-a  20-3-40-<3-15-20-a | 197.3 ± 0.6  (0.70; 53)  190.8 ± 0.7  (0.19; 43) |
| JJJD_43*  JJJD_44  JJJD_41* | 6714881  6714881  6705477 | 276743  276743  280738 | Dioritic intrusions, north of La Serena city | Mdqz (Amp + Bt + Px)  Mdqz (Amp + Px + Bt)  Dqz (Amp + Bt ± Px) | Seriated, euhedral to subhedral (~1 mm, 15 to < 0.5 mm)  Faneritic, equigranular, subhedral (~0.5 mm)  Faneritic, inequigranular, subhedral to anhedral (~0.5 mm, 5 to < 0.2) | 5-16-37-7-20-15-a  5-15-35-10-25-10-a  3-<3-40-5-30-20-a | 155.1 ± 0.6  (0.55; 31)  151.0 ± 0.8  (0.54; 32)  120.6 ± 0.7  (0.64; 45) |
| JJJD_59* | 6884433 | 312480 | San Antonio Diorite | Dt (Px + Amp) | Faneritic, inequigranular, euhedral to anhedral (~5 mm, 15 to < 1) | <3-a-55-20-20-<3-a | 146.2 ± 1.6  (0.28; 20) |
| JJJD_70*  JJJD_57* | 6852201  6876286 | 305182  320753 | Infiernillo | Gdt (Amp + Bt)  Gdt (Amp + Bt ± Px) | Faneritic, inequigranular, euhedral to anhedral (~2 mm, 10 to < 0.5)  Faneritic, inequigranular, euhedral to subhedral (~1 mm, 5 to < 0.2) | 25-15-30-<1-12-18-a  18-10-30-<3-20-20-a | 136.3 ± 0.7  (0.76; 30)  128.4 ± 1.1  (0.38; 29) |
| JJJD_56* | 6866336 | 332867 | La Jaula Diorite | Gdt (Amp + Px) | Faneritic, inequigranular, subhedral (~10 mm, 30 to < 1) | 23-15-47-5-10-a-a | 127.2 ± 0.8  (0.37; 31) |
| JJJD_48*  JJJD_47* | 6742145  6742145 | 288393  288393 | El Trapiche | Gdt (Bt + Amp)  Mg (Bt + Amp) | Seriated, euhedral to anhedral (~3 mm, 10 to < 0.5 mm)  Faneritic, equigranular, euhedral to anhedral (~5 mm) | 20-20-25-a-15-20-a  23-20-25-a-12-20-a | 117.1 ± 0.5  (1.2; 33)  115.9 ± 1.2  (0.15; 35) |
| JJJD_55* | 6836763 | 339725 | Jilguero Granodiorite | Gdt (Amp + Bt) | Faneritic, inequigranular, euhedral to subhedral (~2 mm, 20 to < 0.5) | 25-15-35-a-15-10-a | 95.9 ± 1.3  (0.37; 9) |
| JJJD_77  JJJD_78*  JJJD_76*  JJJD_79*  JJJD_54* | 6820818  6820818  6820818  6820818  6815405 | 327209  327209  327209  327209  326156 | Camarones | Dt (Amp + Bt + Px)  Gdt (Bt + Amp)  Gdt (Bt + Amp)  Gdt (Bt + Amp)  Gdt (Bt + Amp) | Faneritic, inequigranular, euhedral to subhedral (~15 mm, 30 to < 5)  Seriated, euhedral to anhedral (~1 mm, 5 to < 0.2 mm)  Faneritic, inequigranular, euhedral to subhedral (~5 mm, 15 to < 1)  Faneritic, equigranular, euhedral to subhedral (~10 mm, 20 to < 2)  Faneritic, inequigranular, euhedral to subhedral (~15 mm, 25 to < 5) | 10-3-40-12-20-15-a  18-10-37-a-15-20-a  30-25-35-a-5-15-a  20-10-35-a-15-20-a  22-13-35-a-10-20-a | 97.3 ± 0.7  (0.20; 27)  97.3 ± 0.5  (0.33; 35)  96.2 ± 1.7  (0.24; 31)  95.1 ± 0.4  (0.15; 34)  94.8 ± 1.2  (1.5; 23) |

*: Indicates samples analysed for zircon U-Pb geochronology/trace element and whole-rock geochemistry.

Abbreviations: Qz, quartz; Alkfs, alkali feldespars; Pl, plagioclase; Px, pyroxene; Amp, amphibole; Bt, biotite; Msc, muscovite; Opx, orthopyroxene; Clpx, clinopyroxene; Mg, monzogranite; Sg, sienogranite; Gn, granite; Gdt, granodiorite; Tn, tonalite; Dt, diorite; Gb, gabbro; Dqz, quartz diorite; Md, monzodiorite; Mqz, quartz monzonite; Mdqz, quartz monzodiorite. a: absent.

| **Supplementary Table 3.** Zircon and whole-rock petrogenetic indicators by plutonic episode. | | | | | | | |
| --- | --- | --- | --- | --- | --- | --- | --- |
|  |  | **Late**  **Triassic** | **Early**  **Jurassic** | **Late**  **Jurassic** | **Valanginian to**  **early Aptian** | **late Aptian**  **to**  **early Albian** | **late Albian**  **to Cenomanian** |
|  |  | **215-203** | **200-185** | **160-145** | **138-121** | **120-108** | **103-94** |
| **La_N_/Yb_N_**  (crustal thickness) | Median | 3.74 | 5.02 | 4.17 | 3.42 | 6.67 | 6.23 |
|  | P5% – P95% | 1.91 – 8.84 | 1.80 – 9.36 | 2.30 – 8.36 | 1.57 – 6.69 | 4.66 – 8.92 | 3.23 – 13.54 |
| **La/Sm**  (sediments) | Median | 2.96 | 3.63 | 3.11 | 3.39 | 3.68 | 4.54 |
|  | P5% – P95% | 0.60 – 5.78 | 0.35 – 7.18 | 2.18 – 5.36 | 2.03 – 6.50 | 2.76 – 4.93 | 2.69 – 6.40 |
| **Ba/La**  (slab-derived fluids) | Median | 16.7 | 17.4 | 19.4 | 20.8 | 23.6 | 36.8 |
|  | P5% – P95% | 8.7 – 23.4 | 9.3 – 114.2 | 4.9 – 31.6 | 8.9 – 34.0 | 16.5 – 46.2 | 11.7 – 56.9 |
| **Nb/Zr**  (source enrichment) | Median | 0.0635 | 0.0572 | 0.0357 | 0.0249 | 0.0271 | 0.0411 |
|  | P5% – P95% | 0.0334 – 0.4967 | 0.0267 – 0.1591 | 0.0218 – 0.0762 | 0.0035 – 0.0556 | 0.0061 – 0.0655 | 0.0125 - 0.0888 |
| **log fO_2_**  (ΔFMQ) | Median | -2.25 | -1.08 | -2.15 | -2.43 | -2.75 | -1.58 |
|  | Min – Max* | -3.46 – -0.50 | -3.81 – -1.01 | -2.63 – -1.26 | -3.07 – -1.15 | -3.18 – -0.14 | -2.28 – -0.74 |
| **Zircon Th/U**  (extension-compression) | Median | 0.57 | 0.68 | 0.89 | 0.72 | 0.72 | 0.61 |
|  | P5% – P95% | 0.36 – 0.91 | 0.41 – 0.99 | 0.41 – 1.21 | 0.39 – 1.52 | 0.43 – 1.49 | 0.39 – 1.48 |
| **Ti (ppm)**  (Ti-in-zircon temp) | Median | 5.6 | 8.7 | 14.8 | 9.1 | 11.7 | 7.5 |
|  | P5% – P95% | 2.2 – 13.8 | 4.3 – 12.9 | 6.7 – 36.1 | 4.6 – 17.9 | 5.3 – 30.1 | 3.7 – 17.4 |
| **Zircon U/Yb**  (source enrichment) | Median | 0.41 | 0.24 | 0.60 | 0.31 | 0.79 | 0.31 |
|  | P5% – P95% | 0.19 – 1.47 | 0.13 – 0.48 | 0.19 – 2.30 | 0.15 – 1.37 | 0.42 – 1.90 | 0.16 – 0.61 |
| **Zircon Eu_N_/Eu***  (redox state) | Median | 0.36 | 0.26 | 0.11 | 0.19 | 0.19 | 0.52 |
|  | P5% – P95% | 0.04 – 0.56 | 0.18 – 0.49 | 0.06 – 0.51 | 0.06 – 0.36 | 0.08 – 0.43 | 0.14 – 0.71 |
| **Zircon Ce/Nd**  (redox state) | Median | 7.2 | 6.9 | 3.8 | 8.9 | 6.0 | 10.7 |
|  | P5% – P95% | 2.2 – 23.9 | 2.5 – 18.7 | 1.2 – 12.6 | 2.6 – 38.0 | 1.3 – 16.2 | 2.9 – 48.0 |
| Notes: oxygen fugacity series has a limited number of samples with zircon and whole-rock analyses by plutonic episode. Thus, minimum and maximum values are reported instead of percentile 5% and 95%. | | | | | | | |
|  | | | | | | | |

**Supplementary Figure Legends**

**Supplementary Figure 1. Plutonic complexes of the early Andean Cordillera of northern Chile.** Distribution of plutonic complexes in the study area. Samples and their U-Pb zircon ages are indicated, including the data from Supplementary Data 1 and Supplementary Data 2. **AFS**, Atacama Fault System.

**Supplementary Figure 2. Whole-rock classification diagrams.** **a**, total alkali versus silica classification plot^17^, including the alkaline and sub-alkaline limit^18^. **b**, SiO_2_ vs K_2_O diagram^19^, showing shoshonitic and high-, mid- and low-K fields. **c**, SiO_2_ vs FeO*/(FeO*+MgO) plot^20^, defining the ferroan and magnesian fields. **d**, molar A/CNK versus molar A/NK plot^21^, showing the metaluminous, peraluminous and peralkaline fields. **a-d** include the data in Supplementary Data 1, Supplementary Data 2 and Supplementary Data 4; symbol colours refer to the age of the plutonic complexes.

**Supplementary Figure 3.** **Whole-rock Sr/Y ratio**. Sr/y ratio to estimate crustal thickness^22^. Sr/Y ratio plotted according to the outcrop age. Data source: triangles (this study: Supplementary Data 1); circles (ref. 22: Supplementary Data 2), rhomboids (compiled whole-rock analyses: Supplementary Data 4). Symbol colours refer to the age of the plutonic complexes as in Figure 1. Rhomboidal white symbols and lower and upper dotted black lines represent median, 5th and 95th percentiles of each series (kernel density estimation with 2.5 Ma bandwidth and 5 Ma bin width, except for calculated oxygen fugacity). **LT**, Late Triassic (215-203 Ma). **EJ**, Early Jurassic (200-185 Ma). **LJ**, Late Jurassic (160-145 Ma). **V-EAp**, Valanginian to early Aptian (138-121 Ma). **LAp-EAlb**, late Aptian to early Albian (120-108 Ma). **LAlb-Cnm**, late Albian to Cenomanian (103-94 Ma). Colour bands are according to Figure 1. **NVE**, La Negra volcanic event (~180-155 Ma), is represented by the dashed grey band.

**Supplementary Figure 1. Plutonic complexes of the early Andean Cordillera of northern Chile.** Distribution of plutonic complexes in the study area. Samples and their U-Pb zircon ages are indicated, including data from Supplementary Data 1 and Supplementary Data 2. **AFS**, Atacama Fault System.

**Supplementary Figure 2. Whole-rock classification diagrams.** **a**, total alkali versus silica classification plot^17^, including the alkaline and sub-alkaline limit^18^. **b**, SiO_2_ vs K_2_O diagram^19^, showing shoshonitic and high-, mid- and low-K fields. **c**, SiO_2_ vs FeO*/(FeO*+MgO) plot^20^, defining the ferroan and magnesian fields. **d**, molar A/CNK versus molar A/NK plot^21^, showing the metaluminous, peraluminous and peralkaline fields. **a-d** include the data in Supplementary Data 1, Supplementary Data 2 and Supplementary Data 4; symbol colours refer to the age of the plutonic complexes.

**Supplementary Figure 3.** **Whole-rock Sr/Y ratio**. Sr/y ratio to estimate crustal thickness^22^. Sr/Y ratio plotted according to the outcrop age. Data source: triangles (this study: Supplementary Data 1); circles (ref. 22: Supplementary Data 2), rhomboids (compiled whole-rock analyses: Supplementary Data 4). Symbol colours refer to the age of the plutonic complexes as in Figure 1. Rhomboidal white symbols and lower and upper dotted black lines represent median, 5th and 95th percentiles of each series (kernel density estimation with 2.5 Ma bandwidth and 5 Ma bin width, except for calculated oxygen fugacity). **LT**, Late Triassic (215-203 Ma). **EJ**, Early Jurassic (200-185 Ma). **LJ**, Late Jurassic (160-145 Ma). **V-EAp**, Valanginian to early Aptian (138-121 Ma). **LAp-EAlb**, late Aptian to early Albian (120-108 Ma). **LAlb-Cnm**, late Albian to Cenomanian (103-94 Ma). Colour bands are according to Figure 1. **NVE**, La Negra volcanic event (~180-155 Ma), is represented by the dashed grey band.
